# Effects of sealing the intramedullary femoral canal in total knee arthroplasty: A randomized study

**DOI:** 10.1097/MD.0000000000007388

**Published:** 2017-07-21

**Authors:** Xu Li, Xiang-bei Qi, Xue Han, Wei Wang, Jian-ning Liu, Ji-chao Guo, Zhi-yong Li

**Affiliations:** a Department of Orthopedics, The Third Hospital of Hebei Medical University; b Hebei Sport Science Institute, Shijiazhuang, Hebei Province, People's Republic of China.

**Keywords:** arthroplasty, blood loss, femoral canal, intramedullary, knee

## Abstract

**Background:**

This study investigates the clinical effects of sealing the femoral canal by intramedullary alignment instrumentation in total knee arthroplasty (TKA).

**Methods:**

One hundred twenty consecutive patients with knee osteoarthritis, who underwent unilateral TKA, were enrolled in the study and equally randomized into 2 groups: the sealing group and the control group. In the sealing group, the femoral canal was sealed with autogenous bone and cement using intramedullary alignment instrumentation, while the femoral hole was left open for patients in the control group. Blood loss, hemoglobin (Hb) reduction, and other parameters were recorded, as well as the duration of hospital stay and complications. The Hospital for Special Surgery (HSS) knee score was used to assess knee function at the final follow-up appointment.

**Results:**

The calculated blood loss, hidden blood loss, transfusion requirements, drainage volume, and Hb reduction measurements were significantly different (*P* < .05) between the 2 groups. There were no significant differences in the surgery time, intraoperative blood loss, length of hospital stay, HSS score or complications between the 2 groups (*P* > .05).

**Conclusions:**

Sealing the intramedullary canal with autologous bone and a cement plug is an effective method for reducing blood loss and decreasing blood transfusion requirements during TKA procedures that have increasing complication rates.

## 1 Introduction

Total knee arthroplasty (TKA) is a very successful surgical procedure used to treat end-stage knee osteoarthritis to relieve pain and restore joint function.^[1]^ However, TKA can be associated with considerable (1000–1790 mL) blood loss in the perioperative period, which usually requires allogeneic blood transfusion.^[2]^ Blood transfusion can lead to severe complications and increased medical costs. Although various blood-preservation protocols have been created to prevent blood loss, blood transfusion is often required to correct anemia.^[3,4]^

Many surgeons routinely seal the femoral hole in TKA intramedullary alignment to decrease blood loss. Recently, studies have reported that sealing the femoral hole in TKA can prevent hemoglobin (Hb) reduction, postoperative drainage, or blood transfusions.^[5,6]^ However, these studies had limitations such as small sample sizes, inaccurate evaluations, and deficiencies in the study design. Consequently, the efficacy of sealing the femoral hole in TKA remains controversial. The aim of the present study was to evaluate the efficacy and safety of sealing the femoral hole in primary unilateral TKA using a randomized, prospective, comparative study.

## 2 Materials and methods

### 2.1 Study design

This study was approved by the ethics committee of the hospital, and written informed consent was obtained from all participants. We conducted a prospective, randomized clinical study between May 2015 and December 2015. The study was conducted according to the “CONSORT statement” guidelines for randomized controlled trials.

### 2.2 Study subjects

The inclusion criteria used were as follows: patients > 60 years old, diagnosed with osteoarthritis and with a body mass index (BMI) <40 kg/m^2^. The exclusion criteria used were as follows: history of knee trauma or knee surgery, inflammatory polyarthritis, thromboembolic disease, or hemorrhagic blood disease; Hb < 80 g/L; presence of neuromuscular disease, cancer, or metabolic bone disease; or an American Society of Anesthesiologists anesthesia rating > 3.

One hundred twenty patients were allocated to either the autologous bone sealing group or the control group using a random number table, which was computer-generated. This process was conducted by researchers who were blinded to the evaluation of the results. Randomization was conducted using sealed, opaque envelopes and occurred at the time of skin incision in TKA surgery. All patients, anesthesiologists, and the assessor who assessed patients before and after the surgery were blinded as to whether autologous bone sealing was or was not applied.

### 2.3 Surgical procedure

All surgeries were performed by the same skilled surgical team and under spinoepidural anesthesia. A medial parapatellar approach was used to expose the knee joint. An inflatable tourniquet was attached to the limb with a pressure of 100 to 150 mm Hg above the systolic blood pressure. An intramedullary alignment jig was used for the distal femoral resection, and an extramedullary device was used for the tibia. The implant used was a posterior cruciate ligament substituting, cemented total knee prosthetic component (GENESIS II, Smith & Nephew, Inc., Memphis, TN) with no patellar resurfacing. In the treatment group, the femoral hole was sealed with an autologous bone plug obtained from bone off-cuts and cement. In the control group, the femoral hole was left unsealed. Intraarticular drainage was used in all cases. Subcutaneous skin closure was then performed. The tourniquet was deflated until application of a compressive elastic bandage.

A dose of 0.6 mL of low-molecular-weight heparin calcium was administered subcutaneously to all patients at 12 hours postsurgery, and this was repeated daily until discharge. The drainage tube was removed 24 to 48 hours postoperatively in both groups. Active isometric quadriceps movements, initiative straight leg raises, and extension–flexion motions were encouraged 48 hours postoperatively, and ambulation with partial weight bearing was permitted under the supervision of a physical therapist. Patients were cleared for transfusion if their Hb levels dropped to below 80 g/L with symptoms of syncope, fatigue, and/or palpitations.

### 2.4 Follow-up assessment

Patients’ Hb levels were measured preoperatively and at 48 hours postoperatively. Based on the maximum perioperative decrease in the Hb level, total blood loss was calculated according to Gross.^[7]^ The following equation was used for the calculation of total red blood cell loss: total red blood cell loss = preoperative circulating blood volume × ([preoperative hematocrit-postoperative hematocrit]/mean hematocrit). The preoperative circulating blood volume was calculated using the method of Nadler et al as follows: preoperative circulating blood volume = k1 × height (m) + k2 × weight (kg) +  k3 (K is a constant: k1 = 0.3669, k2 = 0.03219, k3 = 0.6041 for males and 0.3561, 0.03308, 0.1833 for females, respectively). Hidden blood loss (HBL) was calculated by subtracting the intraoperative blood loss, drainage volume, and postoperative wound blood loss (obtained by weighing soiled dressings) from the calculated blood loss (CBL) at the 48th postoperative hour. The Hospital for Special Surgery (HSS) knee score was measured preoperatively and at 6 months postoperatively. All complications were recorded, including hematoma, wound infection, and deep vein thrombosis (DVT). Any clinical suspicion of DVT was investigated by Doppler ultrasound. Data collection was then conducted by doctors who were blinded to the study purposes.

### 2.5 Statistical analysis

The sample size calculation was performed based on the difference in the perioperative change in Hb determined from a review of the literature. Sixty patients from each group were required to reach the required power (0.8) when statistical analysis was performed at the level of 2-tailed alpha (0.05).

Statistical analysis was performed with SPSS v. 17.0 statistical software (SPSS, Inc., Chicago, IL). Continuous data are expressed as the mean ± standard deviation and were tested by Student *t* test for differences. Categorical data were analyzed using Chi-square tests. A 2-sided *P* < .05 was considered statistically significant.

## 3 Results

**Figure 1:**
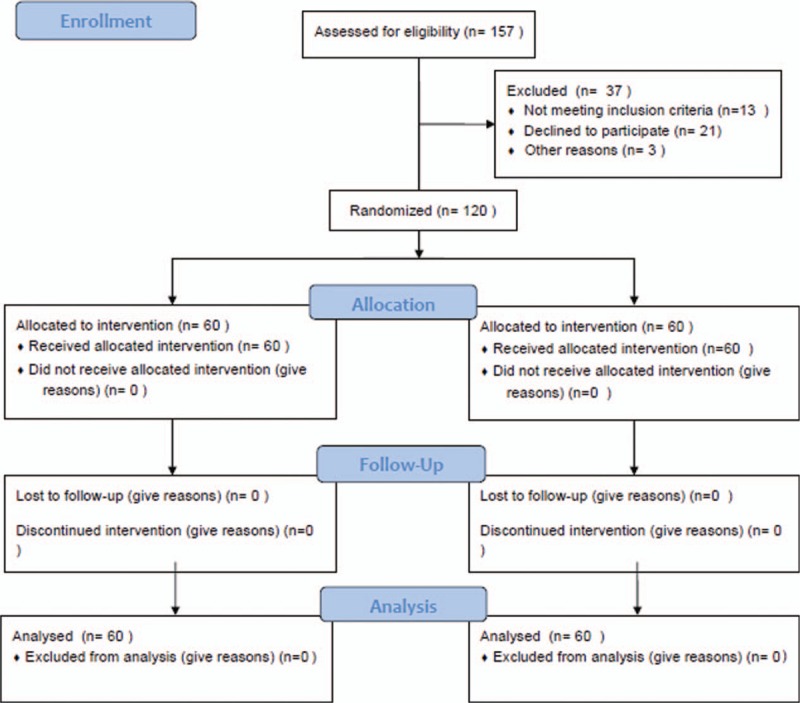
Study flow chart.

The progression of patients through the study is illustrated in Fig. 1. A total of 120 patients met the inclusion criteria for enrollment in the study. Of the 120 patients in the study, 60 were randomized to the treatment group, and 60 were randomized to the control group. There were no significant differences in the baseline patient characteristics between the 2 groups. Demographic data are listed in Table 1.

**Table 1:**
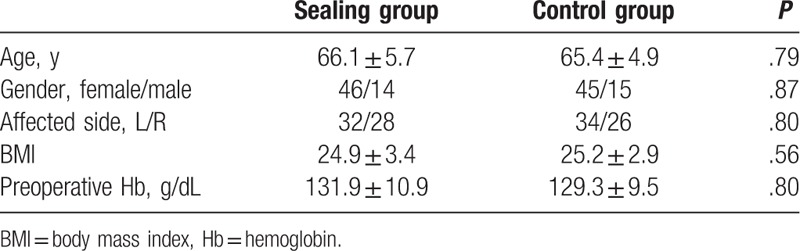
Demographics and baseline measurements.

Intraoperative parameters, such as the surgery time and intraoperative blood loss, were similar between the 2 groups. All operative details are found in Table 2. We observed a significant difference in the CBL, HBL, transfusion requirements, and Hb reduction between the 2 groups (*P* < .05). The HSS values were similar at the 1 year follow-up.

**Table 2:**
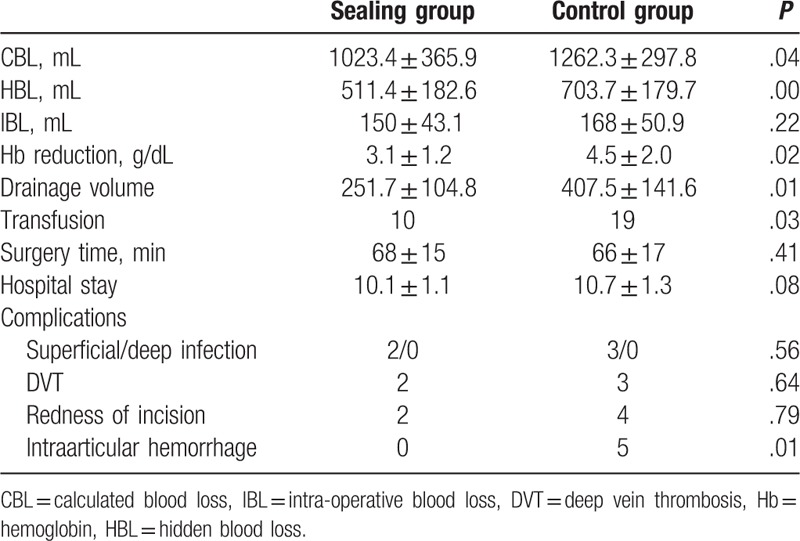
Outcomes and complications.

In terms of adverse events, 2 DVTs were recorded in the treatment group and 3 in the control group. Two superficial infections and 2 erythematous incisions were observed in the treatment group. Three superficial infections and four erythematous incisions were recorded in the control group. No statistically significant differences between the 2 groups were found for DVT, superficial infection, or erythematous incisions. In the control group, 5 patients with recurring intraarticular hemorrhages were observed for 2 months postoperation. All results of bacterial cultures were negative. All patients were treated by articular cavity puncture, suction, and compression bandaging.

## 4 Discussion

The intramedullary alignment system for placing the femoral component was more accurate and practical than extramedullary guides in TKA.^[8]^ However, the intramedullary alignment system requires intramedullary rod damage to cancellous bone and the intramedullary vasculature.^[9]^ In patients with osteoporosis or femoral deformities, this hole could increase the risk of a distal femur fracture and postoperative blood loss. The aim of the present study was to examine the effect of sealing the femoral hole in TKA. Our results showed that sealing the femoral hole in TKA reduced total blood loss, postoperative blood loss, and transfusion requirements.

TKA can be associated with considerable blood loss (1000–1790 mL) in the perioperative period, which usually then requires an allogeneic blood transfusion. In TKA, blood loss is mostly associated with the process of tibial-femoral resected surface preparation and soft tissue release. Dominant blood loss consists of intraoperative blood loss and postoperative drainage volume. Many studies have shown that the application of a tourniquet decreases intraoperative blood loss in TKA,^[10,11]^ and the topical application of tranexamic acid reduces the drainage volume after TKA.^[12–14]^ However, dominant blood loss should not be underestimated when volumes can range from 380 to 800 mL.

Intraoperative blood loss in TKA mainly occurs from the bone cutting surface and the hole of the femoral medullary cavity. After prosthesis installation was completed, the bare bone cutting surface was very small. Therefore, the opening of the femoral hole is the main source of postoperative blood loss. Recent studies have shown that sealing the femoral hole decreases the postoperative drainage volume, while the volume of intraoperative blood loss is similar between the 2 groups. In previous studies, Kumar et al^[6]^ observed that sealing the femoral canal with a bone plug effectively reduced blood drainage. Ko et al^[5]^ reported opposite results. We sealed the femoral canal with autologous bone and a cement plug, which was more effective than the bone plug used in the aforementioned studies.

Studies have reported that HBL accounts for more than 50% of the total blood loss in TKA. Blood loss from an unsealed femoral hole also leads to more hidden hemorrhaging. None of the previous studies referred to the effect of sealing the femoral hole on HBL in TKA.^[5,6,15]^ In the present studies, sealing the femoral hole significantly decreased HBL (511.4 mL vs 703.7 mL, *P* < .05).

Although various blood-preservation protocols have been created to prevent blood loss, some patients still require blood transfusions to correct anemia. In the present study, the total rate of blood transfusion was similar to that found in published studies. Sealing the femoral hole decreased transfusion requirements significantly (10% vs 19%).

We found 5 patients with recurrent intraarticular hemorrhage during the 2 months postoperation in the control group but none in the sealing group. All results of the bacterial cultures were negative. Patients were cured by articular cavity puncture, suction, and compression bandaging. Our results showed that sealing the femoral hole did not increase the incidence of DVT, superficial infection, or erythema of the incision.

Some limitations of this study should not be ignored. First, we conducted only a short follow-up, including limited parameters. Long-term observation is needed to reach a comprehensive conclusion. Second, the quality of the study may be limited by its methodology. The impossibility of blinding the surgeon to each patient's group may lead to bias. Third, we must be cautious when interpreting the statistical significance of the findings. Further studies are thus required to arrive at a consensus regarding the benefit of sealing the femoral hole in patients undergoing TKA.

## 5 Conclusion

Sealing the intramedullary canal with autologous bone and a cement plug is an effective method for reducing blood loss and decreasing blood transfusion requirements during TKA with increasing complications.
